# Bitter Melon Reduces Head and Neck Squamous Cell Carcinoma Growth by Targeting c-Met Signaling

**DOI:** 10.1371/journal.pone.0078006

**Published:** 2013-10-17

**Authors:** Ananthi Rajamoorthi, Shubham Shrivastava, Robert Steele, Pratibha Nerurkar, Juan G. Gonzalez, Susan Crawford, Mark Varvares, Ratna B. Ray

**Affiliations:** 1 Department of Pathology, Saint Louis University, St. Louis, Missouri, United States of America; 2 Saint Louis University Cancer Center, Saint Louis University, St. Louis, Missouri, United States of America; 3 Laboratory of Metabolic Disorders and Alternative Medicine, Department of Molecular Biosciences and Bioengineering, College of Tropical Agriculture and Human Resources, University of Hawaii, Honolulu, Hawaii, United States of America; Ohio State University, United States of America

## Abstract

Head and neck squamous cell carcinoma (HNSCC) remains difficult to treat, and despite of advances in treatment, the overall survival rate has only modestly improved over the past several years. Thus, there is an urgent need for additional therapeutic modalities. We hypothesized that treatment of HNSCC cells with a dietary product such as bitter melon extract (BME) modulates multiple signaling pathways and regresses HNSCC tumor growth in a preclinical model. We observed a reduced cell proliferation in HNSCC cell lines. The mechanistic studies reveal that treatment of BME in HNSCC cells inhibited c-Met signaling pathway. We also observed that BME treatment in HNSCC reduced phosphoStat3, c-myc and Mcl-1 expression, downstream signaling molecules of c-Met. Furthermore, BME treatment in HNSCC cells modulated the expression of key cell cycle progression molecules leading to halted cell growth. Finally, BME feeding in mice bearing HNSCC xenograft tumor resulted in an inhibition of tumor growth and c-Met expression. Together, our results suggested that BME treatment in HNSCC cells modulates multiple signaling pathways and may have therapeutic potential for treating HNSCC.

## Introduction

Head and neck squamous cell carcinoma (HNSCC) is the sixth most prevalent cancer in the world. Overall survival rate has not significantly improved in the past couple of decades, despite significant improvements in surgical procedures, radiotherapy, and chemotherapy [1]. In the United States, 50,000 new cases are diagnosed, and nearly 10,000 deaths are attributable to this disease annually [1]. HNSCCs are highly heterogeneous and contain a large number of genetic alterations which make them refractory to specific targeted drugs. The epidermal growth factor receptor (EGFR) is overexpressed in ∼90% of the HNSCC, and involved in cell growth, invasion, angiogenesis and metastasis [1], [2]. The c-Met pathway is also aberrantly upregulated in HNSCC, and activates the same downstream signaling pathway as EGFR. The ubiquitous expression of tyrosine kinase, such as EGFR and/or c-Met, is higher in HNSCC tumors, however, the clinical response rate using these tyrosine kinase inhibitors is limited due to intrinsic and acquired resistance [3]. Therefore, new approaches are necessary to further reduce the mortality of this disease.

One approach is to treat HNSCC through dietary means. Natural products are non-toxic and offer promising options for developing effective chemotherapeutics either alone or in combination with existing therapy. Bitter melon (*Momordica charantia*) is widely cultivated in Asia, Africa, and South America, and extensively used in folk medicines as a remedy for diabetes, specifically in South East Asia. Animal studies have employed either fresh bitter melon extract (BME) or crude organic fractions to evaluate its beneficial effects on glucose metabolism and on plasma and hepatic lipids [4], [5]. We have previously shown that BME (without seeds) treatment of human cancer cells induced cell cycle arrest by altering critical signaling molecules and impairing cell growth [6], [7]. BME feeding also prevented high grade prostatic intraepithelial neoplasia formation in a TRAMP mouse model [7]. Antiproliferative activity of bitter melon extract was recently noted in other cancer cell lines [8]–[10]. However, the anti-cancer potential of BME against HNSCC and its underlying mechanism has not been explored. In this study, we have examined the effect of BME in a number of HNSCC cell lines. We have demonstrated for the first time that BME treatment inhibits c-Met expression and its downstream signaling pathway. Further, we have observed that BME feeding in mice regresses the HNSCC xenograft tumor growth, suggesting its potential as a therapeutic agent against HNSCC.

## Materials and Methods

### Cell Lines

Cal27 cells (tongue, mutant p53) were recently obtained from ATCC. JHU-22 (larynx, wild type of p53) and JHU-29 (tongue, wild type of p53) cells were recently procured from the Johns Hopkins University [11], [12]. Cal27 and JHU-22 cells were maintained in Dulbecco's Modified Eagle Medium (Sigma) supplemented with 10% FBS and 1% penicillin/streptomycin (Sigma). JHU-29 cells were maintained in RPMI-1640 Medium (Sigma) supplemented with 10% FBS and 1% penicillin/streptomycin. Cal27, JHU-22 and JHU-29 cells were treated with different doses of BME, and untreated cells were used in parallel as controls. BME was prepared from the Chinese variety of young bitter melons (raw and green) as discussed previously [6]. Briefly, BME was extracted using a household juicer and centrifuged at 560 x *g* at 4°C for 30 min, freeze dried at -45°C for 72 h and stored at −80°C until used for feeding studies. We prepared a stock of 0.1 g/ml in water, aliquoted, and used for *in vitro* cell culture work and 100 µl/mouse for oral gavage.

### Cell proliferation assay

Trypan blue exclusion method was used to investigate cell proliferation in control and BME treated Cal27 cells. Live cells were counted using a hemocytometer (Fisher Scientific) at different time points. MTS assay (Promega) was also used for cell viability assay.

### Human Cell Cycle Array

RNA was isolated from control and BME treated Cal27 cells. A RT^2^ profiler PCR Array for human cell cycle (Qiagen Inc., PAHS-020Z) was performed as described previously [13]. Array data was analyzed using free web based software http://pcrdataanalysis.sabiosciences.com/PCR/arrayanalysis.php and automatically perform all ΔΔC_t_ fold change calculations.

### Xenograft tumor growth assay

Cal27 cells were trypsinized, washed, and resuspended in serum free Dulbecco's Modified Eagle Medium. 2×10^6^ (100 µl) cells containing 40% BD-Matrigel were injected subcutaneously into the flank of five week old BALB/c athymic nude mice (Harlan Laboratories). When tumor volume reached ∼60 mm^3^, mice were randomly divided in two groups. One group received 100 µl of BME by gavage daily for 5 days/week and the other group received 100 µl of ddH_2_0 by gavage for control, as described previously [7]. BME dosage was selected based on our previous study [7]. Tumors were measured using a slide Caliper once a week and volume was calculated using the formula L x H x W x 0.5236, as described previously [14], [15]. After 4 weeks of treatment, mice were sacrificed; tumors were dissected and divided into two groups. In one group, tumors were fixed in formalin and processed for H & E staining and immunohistochemistry. The other group of tumors was snap frozen for biochemical analysis.

### Ethics statement

The animal experiments are conducted using highest standards for animal care in accordance with the NIH Guidelines for the Care and Use of Laboratory Animals, and approval of Saint Louis University Animal Care Committee (Approval number: 1017).

### Western Blotting

Cell lysates were prepared from control or BME treated Cal27, JHU-22 and JHU-29 cells for Western blot analysis using specific antibodies. Protein lysates were also prepared from collected tumor tissues of control or BME treated Cal27 xenograft mice. Proteins were separated by SDS-PAGE and transferred onto 0.45 µM nitrocellulose membrane. Membranes were blocked using 5% low fat dry milk in TBST and probed with the following primary antibodies. Proteins were detected using ECL Western Blotting Substrate (Thermo Scientific) and autoradiography. Protein loads were normalized using antibodies for GAPDH (Cell Signaling Technologies) or tubulin (Santa Cruz Biotechnology). PCNA expression level was examined from control and BME-fed mice by immunohistochemistry (IHC). The following antibodies were used in this study: c-Met, c-myc, Stat3, phospho-Stat3 (Tyr 705), Mcl-1, cleaved caspases 3 and 9, PARP (Cell Signaling Technologies), and Cyclin D1 (Santa Cruz Biotechnology).

### Statistical analysis

Two-tailed Student's *t*-test was used for statistical analysis, as described previously [14], [15].

## Results

### BME treatment in HNSCC inhibits cell proliferation

**Figure 1:**
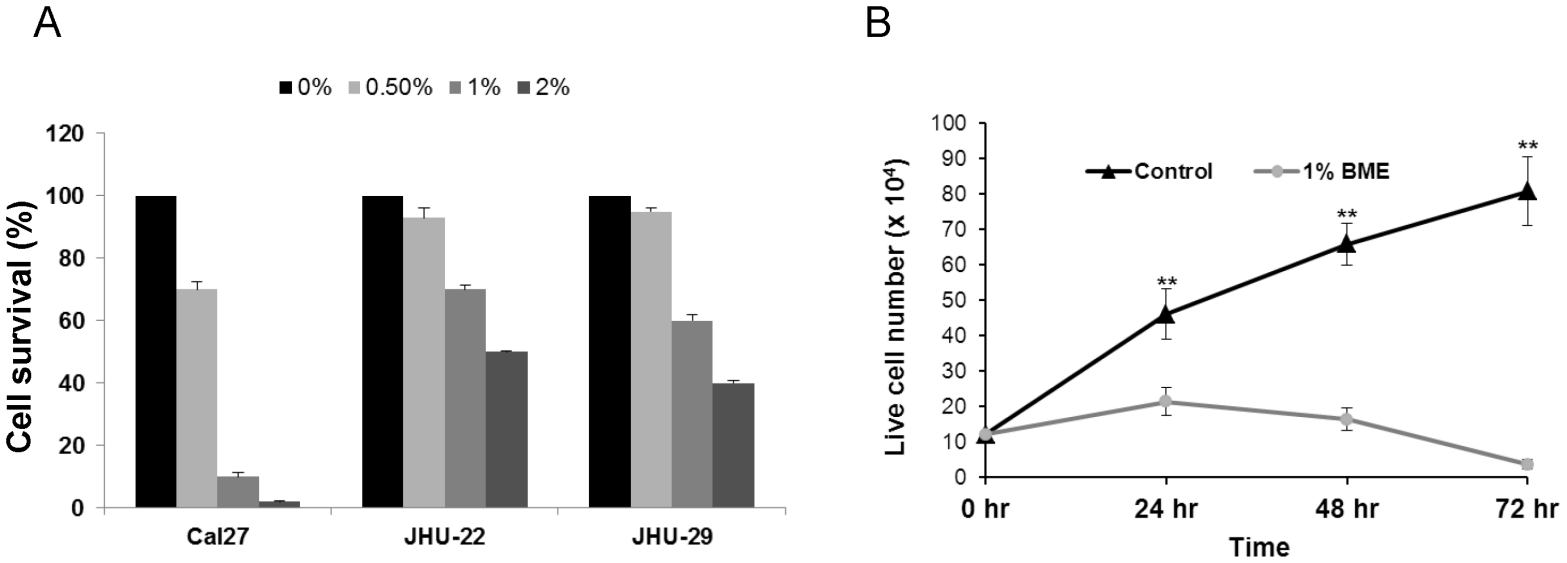
BME treatment in HNSCC cells inhibits proliferation. (A). HNSCC cells were treated with BME for 24 h, and MTS assay was performed. (B). Cal27 cells were treated with BME for different time points. Cell proliferation was measured in control and BME treated Cal27 cells by Trypan blue exclusion method. The results are presented as means of three different experiments with standard errors. (**, p<0.001).

**Figure 2:**
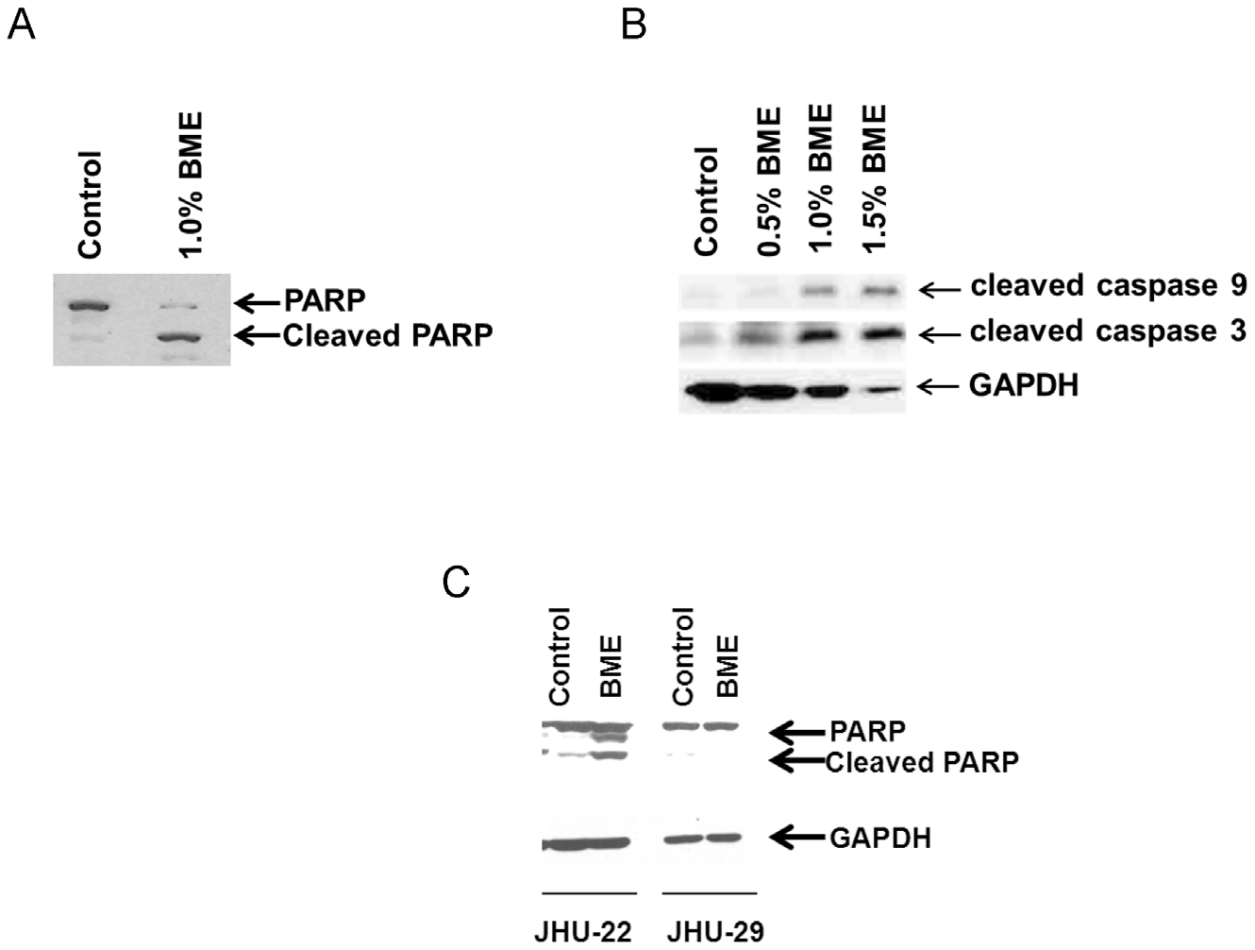
BME treatment in HNSCC cells induced PARP cleavage and caspase activation. (A). Lysates were prepared from Cal27 cells treated or untreated with BME for 72 h, and subjected to Western blot analysis using a specific antibody to PARP. Following treatment of BME, PARP was cleaved to an 86 kD signature peptide. (B). Treatment of Cal27 cells with BME induced caspase 9 and 3 cleavage. The cleaved product of caspase 9 and caspase 3 are shown. The blot was reprobed with an antibody to GAPDH for comparison of equal protein load. (C). JHU-22 and JHU-29 cells were treated with BME and cell lysates were subjected to Western blot analysis for PARP cleavage using specific antibody. The blot was reprobed with an antibody to GAPDH for comparison of equal protein load.

We have shown previously that BME treatment exerts an antiproliferative effect in breast and prostate cancer cells [6], [7]. In this study, we treated HNSCC cancer (Cal27, JHU-22 and JHU-29) cells with BME using different doses, and cell viability was determined by MTS assay. A dose dependent and cell type specific effect was observed (Figure 1A). We further examined Cal27 cell growth following treatment with BME by Trypan blue exclusion. We observed that Cal27 cells treated with 1% BME displayed inhibition of cell proliferation at different time points (Figure 1B). We further examined the effect on erlotinib, a tyrosine kinase inhibitor, as a positive control [16], and observed HNSCC cell death in a dose dependent manner (data not shown). We observed previously that BME treatment in prostate cancer cell lines induced apoptotic cell death [7]. To examine the mechanism of BME mediated cell death in HNSCC cell lines, the apoptotic signaling pathway was investigated by Western blot analysis using specific antibodies. Cal27 cells treated with BME displayed a significant cleavage of the native 116 kD PARP to its 86 kD signature peptide (Figure 2A). We also observed that BME treatment activated caspase 9 and caspase 3 in Cal27 cells (Figure 2B). We next examined the effect of BME in JHU-22 and JHU-29 cell lines. JHU-22 cells following BME treatment induced PARP cleavage (Figure 2C). However, JHU-29 cells treated with BME did not display significant PARP cleavage (Figure 2C), suggesting this cell line is not inducing apoptotic cell death.

### BME suppresses c-Met and the downstream signaling molecules in HNSCC cell lines

**Figure 3:**
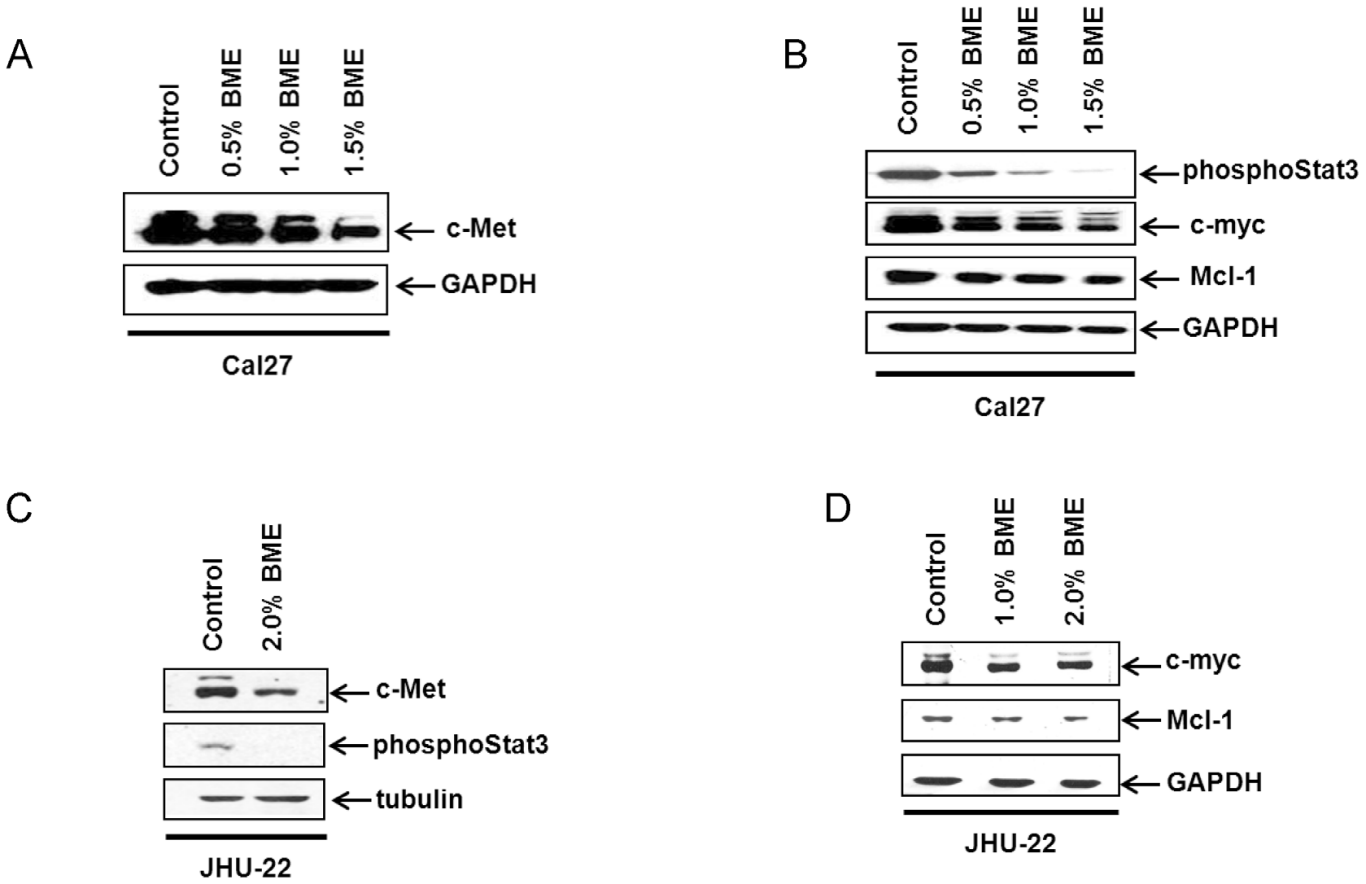
Treatment of BME in HNSCC cells decreases c-Met protein expression and its downstream signaling pathways. (A). Cal27 cells were treated with BME in a dose dependent manner and reduced c-Met expression was noted by Western blot analysis. The blot was reprobed with an antibody to GAPDH for comparison of equal protein load. (B). The expression levels of phosphoStat3 (Tyr705), c-myc and Mcl-1 in control and BME treated Cal27 cells were examined by Western blot using specific antibodies. The blot was reprobed with an antibody to GAPDH for comparison of protein load. (C). JHU-22 cells were treated with BME for 48 h and cell lysates were analyzed for c-Met and phosphoStat3 expression by Western blot analysis using specific antibody. The blot was reprobed with an antibody to tubulin for comparison of protein load. (D). The expression levels of c-myc and Mcl-1 in control and BME treated JHU-22 cells were examined by Western blot using specific antibodies. The blot was reprobed with an antibody to GAPDH for comparison of protein load.

To investigate the effect of BME on c-Met signaling pathway, Cal27 cells were treated with BME, and cell lysates were analyzed by Western blot. We observed reduced c-Met expression (20–50%) in a dose dependent manner (Figure 3A). We next examined the c-Met downstream signaling pathway in BME treated HNSCC cells. The expression of phosphoStat3 (Tyr705) was reduced in BME treated Cal27 cells, and total Stat3 expression levels were unaltered (data not shown) in BME treated Cal27 cells. Our results also suggested that BME treatment in Cal27 cells inhibited c-myc expression level in a dose dependent manner (Figure 3B). Mcl-1 is an antiapoptotic protein in the Bcl-2 family member that is highly regulated in normal cells, and when dysregulated, it contributes to cancer. Enhanced Mcl-1 expression has been observed in multiple human cancers, often in association with poor prognosis, disease recurrence, or drug resistance [17]–[19]. We have also observed that Mcl-1 (long anti-apoptotic form) is inhibited following BME treatment (Figure 3B). Reduced c-Met expression level was also observed in JHU-22 cells following BME treatment as compared to control (Figure 3C). However, no significant change in c-Met expression was noted in JHU-29 cells (data not shown), and did not include for subsequent studies. Further, reduced expression of phosphoStat3 (Tyr705), c-myc and Mcl-1 was also observed in BME treated JHU-22 cells as compared to untreated control (Figure 3C and 3D). We have also observed the downregulation of c-Met expression in Cal27 cells following treatment with 10 µM erlotinib (data not shown).

### BME treatment in HNSCC cells modulates cell cycle regulatory genes

**Figure 4:**
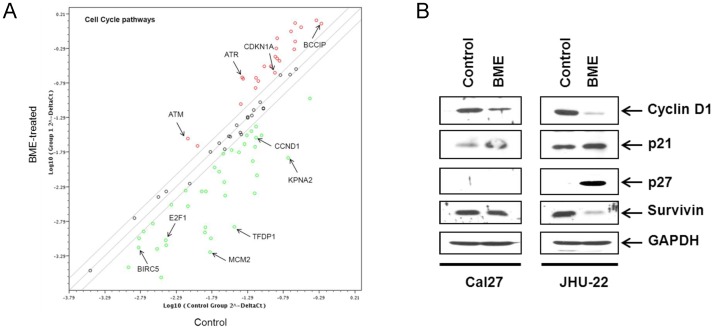
Modulation of cell cycle regulatory molecules following BME treatment in HNSCC cells. (A). Gene expression profiling from RNA of mock or BME treated Cal27 cells was performed using SABiosciences cell cycle pathway qRT-PCR arrays. BME treated cells display a pattern of modulated cell proliferation markers (e.g., ATM, CCND1, MCM2 and BIRC5). (B). Cal27 and JHU-22 cells were treated with BME and cell lysates were analyzed for certain cell cycle molecules for verification. The expression of CyclinD1, p21, p27 and survivin was examined by Western blot using specific antibodies. The blots were reprobed with an antibody to GAPDH for comparison of protein load.

To investigate whether BME mediated cell cycle regulation is associated with the expression of cell-cycle specific regulatory genes, we performed a pathway specific cell cycle array described in Materials and Methods. Our array data suggested that BME treatment in Cal27 cells modulates several cell cycle regulatory proteins (Figure 4A). Among them, upregulation of the possible tumor suppressor gene ataxia telangiectasia mutated (ATM) suggested the activation of negative regulation of cell cycle protein upon BME treatment. Survivin is the smallest member of the inhibitor of apoptosis (IAP) protein family and is encoded by the BIRC5 gene. Cell proliferation is tightly regulated by cyclins and cyclin dependent kinases (Cdks). p21 and p27 are the most common Cdk inhibitor that interacts with cyclins and cause cell growth arrest in several cancerous tissues. Inhibition of survivin (product of gene BIRC5) and activation of p21 (product of gene CDKN1A) and p27 (product of gene CDKN1B) by BME was noted, associating with cell cycle arrest at G2/M phase [20]. Down-regulation of transcription factors E2F1 and DP-1 (product of gene *TFDP1*) was also linked to cell cycle arrest at G2/M phase in cancer cells. KPNA2, a gene overexpressed in many cancers [21], [22], and MCM2 gene, an initiator of replication and necessary for entry into S phase of the cell cycle [23], were downregulated in the human cell cycle array. To further verify the modulation of cell-cycle regulatory molecules, we examined the expression of cyclins and cyclin-dependent kinase (CDK) inhibitors at the protein level. Control and BME treated Cal27 or JHU-22 cell lysates were prepared for Western blot analysis. Our results demonstrated that treatment of BME led to a significant downregulation of cyclin D1 and survivin in Cal27 and JHU-22 cell lines (Figure 4B). We have also observed a significant increase of p21 protein in HNSCC cell lines. Interestingly, p27 upregulation was only noted in BME treated JHU-22 cells. Therefore, the induction of cell cycle arrest of HNSCC cells by BME was associated with the down-regulation of genes involved in G2/M phase.

### Oral administration of BME inhibits HNSCC tumor growth *in vivo* in Xenograft mouse model

**Figure 5:**
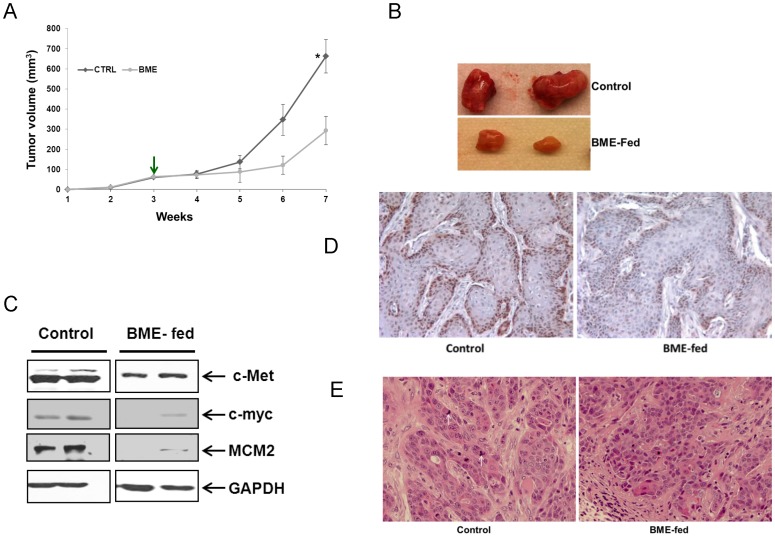
Oral BME administration in Cal27 xenograft nude mice reduces tumor growth. (A). Cal27 cells were implanted subcutaneously into the flank of athymic nude mice. Tumor bearing mice were randomized into two groups, and water (control) or BME was gavaged orally for 4 weeks (5 day/wk). Down arrow indicates the BME or water gavage start time. Volume of tumor growth was monitored once a week and presented as a mean. Small bar indicates standard error (*, p<0.05). (B). Representative tumors dissected from the control and BME-fed mice are shown. (C). Western blot of c-Met, c-myc and MCM2 in xenograft tumor tissues isolated from both control and BME fed mice. The blot was reprobed with an antibody to GAPDH for comparison of protein load. (D). The immunohistochemical staining for PCNA in representative tumor section of a control and BME-fed mice (magnification, 40×) are shown. (E). A representative tumor section from control or BME-fed mice stained with hematoxylin and eosin (magnification, 40×) are shown. Arrows indicate the mitosis.

We next examined whether BME feeding could regress HNSCC xenograft tumor growth. For this, Balb/c athymic nude mice (5 weeks old) were purchased from Harlan Sprague Dawley (Indianapolis, IN). Cal27 cells were grown, harvested, washed with PBS, and resuspended in serum free DMEM. Mice were injected s.c. into each posterior flank region with approximately 2 × 10^6^ cells (100 µl amount) with 40% Matrigel. Tumors were allowed to grow for ∼3 weeks (∼60 mm^3^ tumor volume), and mice were divided into two groups (6 mice/gr). The experimental group was gavaged with BME for five days a week, and control group similarly received water as described previously [7]. After 4 weeks of treatment with BME or water, the mice were sacrificed and tumors were collected. Tumor volume was measured every week for 4 weeks as described previously [14], [15]. A reduction in tumor growth and volume was observed in BME-fed mice as compared to control mice (Figure 5A and 5B). Tumors were collected after sacrificed the mice, and divided into two groups. Half of the tumors were used for histopathology and other half was used for biochemical evaluation.

We have observed that BME treatment in Cal27 cells inhibited c-Met expression *in vitro*. To examine whether BME feeding inhibits c-Met expression in tumors, Western blot analysis was performed using tumor samples. Our data suggested that c-Met and c-myc expression were inhibited in BME-fed mice as compared with control mice (Figure 5C). Since our array data suggested that BME treatment in Cal27 cells modulated cell cycle regulatory molecules, we examined the expression level of MCM2 in both groups. Our results demonstrated that BME feeding inhibited the expression of MCM2 protein *in vivo* (Figure 5C). To further confirm the cell proliferation in tumors from control and experimental groups, immunohistochemistry of PCNA was performed. The proliferation rate of control group was significantly higher as compared to BME-fed mice (Figure 5D). Further, H & E staining suggested the reduction of keratinocyte formation and mitosis in tumor from BME-fed mice, which was reflected with the tumor volume (Figure 5E). Thus, these results indicated that BME mediated c-Met inhibition and modulation of its downstream signaling molecules play a role, in part, for reduction of Cal27 tumor growth.

## Discussion

In this study, we have demonstrated that BME treatment significantly reduces cell proliferation of several human HNSCC cell lines. Aberrant activation of the c-Met pathway has been implicated in the development and progression of head and neck cancer [2], [3], [24], [25]. Numerous antagonists and c-Met inhibitors have been developed to target the HGF/c-Met signaling pathway over recent years [16], [25]. Thus, the c-Met pathway is a potential therapeutic target for HNSCC. Our data suggested that BME treatment in HNSCC cell lines (Cal27 and JHU-22) display significant inhibition of c-Met expression. Subsequent studies demonstrated that BME treatment modulates the downstream molecules of c-Met signaling pathway, such as, phosphoStat3, c-myc, and Mcl-1 in HNSCC cells. However, we did not see significant alteration of EGFR or phosphoAKT expression in BME treated Cal27 cells (data not shown). Apoptosis in response to chemotherapeutic drugs is one of the common mechanisms in cancer cells [26]. We have observed that BME treatment in HNSCC cell lines are inducing apoptotic cell death.

Cell cycle progression plays an important part in tumor growth and its regulation is an effective strategy for the control of tumor growth [20]. Our *in vitro* data indicated that BME treatment of HNSCC cells resulted in downregulation of cyclin D1 and survivin, and upregulation of p21/p27. These data suggested that BME exerts a role in HNSCC cell growth regulation as we observed previously with breast and prostate cancer cells [6], [7]. Together these data suggested that cell cycle regulation is another signaling pathway by which BME exerts anti-tumor effect in HNSCC cells.

We have used an *in vivo* model of tumor xenograft to verify the chemotherapeutic potential of BME against HNSCC cell growth. We have shown previously that BME feeding prevented high grade prostatic intraepithelial neoplasia formation in a TRAMP mouse model without any toxic effect [7]. Here, our results suggested that BME feeding in Cal27 xenograft bearing mice display reduced tumor growth without any apparent sign of toxicity in the athymic nude mice. The identification of molecular targets is important in terms of monitoring the clinical efficacy of cancer therapeutic strategies. Our data strongly demonstrated that upon BME feeding reduced expression of c-Met, c-myc, PCNA and MCM2 as signature molecular targets was observed in Cal27 xenograft tumors.

In summary, our results demonstrated for the first time the chemotherapeutic efficacy of BME on head and neck cancer cell growth *in vitro* and tumor xenograft growth *in vivo* by inhibiting the c-Met signaling pathway. Thus, BME appears to be an attractive dietary product for the management of head and neck cancer. Future studies are indeed necessary using different *in vivo* models to further verify efficacy and identify additional molecular targets of BME for inhibition of HNSCC growth.
